# High Risk of Viral Reactivation in Hepatitis B Patients with Systemic Lupus Erythematosus

**DOI:** 10.3390/ijms22179116

**Published:** 2021-08-24

**Authors:** Ming-Han Chen, Chien-Sheng Wu, Ming-Huang Chen, Chang-Youh Tsai, Fa-Yauh Lee, Yi-Hsiang Huang

**Affiliations:** 1 Division of Allergy-Immunology-Rheumatology, Department of Medicine, Taipei Veterans General Hospital, Taipei 11217, Taiwan; cytsai@vghtpe.gov.tw; 2 Faculty of Medicine, National Yang Ming Chiao Tung University, Taipei 11221, Taiwan; mhchen9@vghtpe.gov.tw; 3 Division of Allergy-Immunology-Rheumatology, Department of Medicine, Far Eastern Memorial Hospital, Taipei 220216, Taiwan; wucs@femh.org.tw; 4 Division of Gastroenterology, Department of Medicine, Taipei Veterans General Hospital, Taipei 11217, Taiwan; fylee@vghtpe.gov.tw (F.-Y.L.); yhhuang@vghtpe.gov.tw (Y.-H.H.); 5 Institute of Clinical Medicine, School of Medicine, National Yang Ming Chiao Tung University, Taipei 11221, Taiwan

**Keywords:** systemic lupus erythematosus, HBV reactivation, immunosuppressive therapy, HBV carrier, resolved hepatitis B

## Abstract

HBV reactivation (HBVr) can occur in hepatitis B surface antigen (HBsAg)-positive and negative patients. Here, we determined the incidence of HBVr and its related hepatitis in patients with systemic lupus erythematosus (SLE). From 2000 to 2017, 3307 SLE cases were retrospectively reviewed for episodes of hepatitis. The incidence, long-term outcomes and risk factors associated with HBVr, including HBsAg reverse seroconversion (RS) were analyzed. Among them, 607 had available HBsAg status. Fifty-five (9.1%) patients were positive for HBsAg and 63 (11.4%) were HBsAg-negative/antibody to hepatitis B core antigen (anti-HBc)-positive (resolved hepatitis B infection, RHB). None of them received antiviral prophylaxis before immunosuppressive treatment. During a mean 15.4 years of follow-up, 30 (54.5%) HBsAg-positive patients developed HBVr and seven (23.3%) died of liver failure, whereas only two (3.2%) RHB cases experienced HBsAg reverse seroconversion (RS). Multivariate logistic regression analysis showed that age ≥ 40 years at diagnosis of SLE (HR 5.30, *p* < 0.001), receiving glucocorticoid-containing immunosuppressive therapy (HR 4.78, *p* = 0.003), and receiving glucocorticoid ≥ 10 mg prednisolone equivalents (HR 3.68, *p* = 0.003) were independent risk factors for HBVr in HBsAg-positive patients. Peak level of total bilirubin ≥ 5 mg/dL during HBVr was an independent factor of mortality (*p* = 0.002). In conclusion, the risk of HBVr was associated with glucocorticoid daily dose. Antiviral prophylaxis is mandatory for SLE patients diagnosed at age of ≥40 years who receive ≥ 10 mg daily dose of oral prednisone or equivalent.

## 1. Introduction

Chronic hepatitis B virus (HBV) infection poses a public health issue because it accounts for significant morbidity and mortality. The prevalence of hepatitis B surface antigen (HBsAg) has been reported as 3.6% worldwide, with a higher prevalence in most Asian countries [1,2]. HBV reactivation (HBVr) could be a life-threatening complication and can occur in patients with rheumatic diseases, cancer, organ transplantation, or those receiving immunosuppressive therapy [3]. The reactivation rate of HBV is estimated to be around 12–24% in HBsAg-positive patients with rheumatic diseases on immunosuppressive therapy [4,5] and increased to 20–50% in those with cancer undergoing chemotherapy [3,6]. Not only in HBsAg-positive patients, HBVr can also develop in HBsAg-negative/antibody to hepatitis B core antigen (anti-HBc)-positive patients (resolved hepatitis B, RHB), resulting in HBsAg reverse seroconversion (RS) [7,8,9,10]. In general, the risk of HBVr in patients with RHB is lower than HBsAg-positive cases. Moreover, the risk of HBVr can happen even long after withdrawal of cytotoxic chemotherapy drugs [7,10,11].

Systemic lupus erythematosus (SLE) is a systemic autoimmune disease that mainly affects women of childbearing age. It is characterized by multiple organ involvement, including kidneys, heart, central nervous system, and lungs, and varies in clinical features, such as skin rashes, arthralgia, or arthritis, hematologic disorders, and serositis [12]. The seropositive rates of HBsAg and anti-HBc have been reported to be low in patients with autoimmune diseases, but the association between HBV infection and SLE remains unclear [13,14,15,16]. Glucocorticoid and hydroxychloroquine play a central role in the treatment of SLE [17]. Other immunosuppressive agents, such as cyclophosphamide, azathioprine, mycophenolate mofetil, etc., are frequently combined and used depending on the disease activity and presenting symptoms to minimize long-term exposure to high-dose glucocorticoid. However, glucocorticoid has been proven to increase HBV DNA replication via binding to the glucocorticoid responsive element [18,19]. Other immunosuppressive agents can potentially increase the risk of HBVr.

Although there is a risk of HBVr under immunosuppressive treatment in HBsAg-positive and RHB patients, the incidence of HBVr in SLE patients over a long period of time and its association with types of immunosuppressants remains unknown. The aim of this study was to delineate the clinical features, outcomes, and risk factors of HBVr in SLE patients who were positive for HBsAg or RHB.

## 2. Results

### 2.1. Hepatitis B Status and Immunologic Profiles of Patients

**Figure 1:**
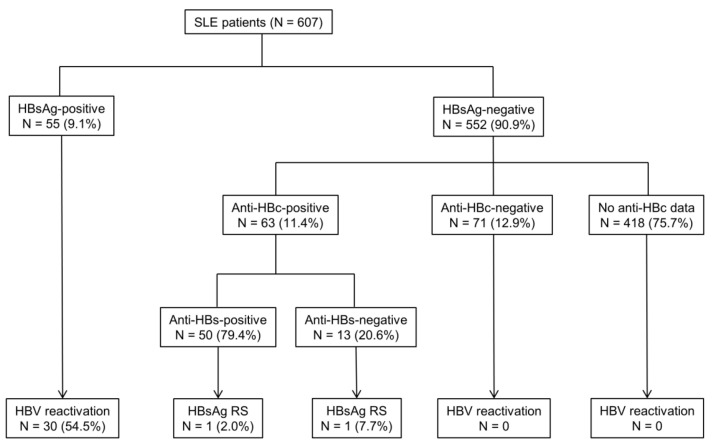
HBV status and the incidence of hepatitis related to HBV reactivation in SLE patients. SLE patients were categorized according to hepatitis B surface antigen (HBsAg), antibody to hepatitis B core antigen (anti-HBc), and antibody to hepatitis B surface antigen (anti-HBs) status. HBV reactivation in HBsAg-positive patients was defined as either an increase in HBV DNA > 1 Log10 IU/mL compared with baseline or HBV DNA > 20,000 IU/mL in cases without baseline HBV viral load after diagnosis or the use of immunosuppressive agents. HBsAg reverse seroconversion (RS) in HBsAg-negative/antibody to hepatitis B core antigen-positive patients was defined as reappearance of HBsAg in the serum.

Among 607 patients who had available HBsAg, 55 (9.1%) were positive for HBsAg and 63 (11.4%) had RHB (Figure 1). None of them received anti-HBV prophylaxis before HBVr, according to Taiwan National Insurance regulation. The mean age at diagnosis of SLE was 37.0 years for HBsAg-positive patients and 33.6 years for RHB patients, and 44 (80.0%) HBsAg-positive patients and 53 (84.1%) RHB patient were female (Table 1). The age at diagnosis of SLE and sex ratio did not significantly differ between the two groups. In HBsAg-positive SLE patients, 21.3% (10 out of 47) were positive for hepatitis B virus e antigen (HBeAg). Among these RHB patients, 50 (79.4%) were antibodies to HBsAg (Anti-HBs)-positive. The immunologic profiles of SLE patients showed high ANA titers (≥1:80) in all patients (100.0%). The frequency of anti-SSA/Ro, anti-smith, and anti-RNP antibodies were higher in RHB patients than in HBsAg-positive patients (all *p* < 0.05). The follow-up period was significantly longer in RHB patients than in HBsAg-positive patients (18.6 ± 10.2 vs. 11.7 ± 9.4 years, *p* < 0.001).

### 2.2. Incidence of HBVr in SLE Patients

**Figure 2:**
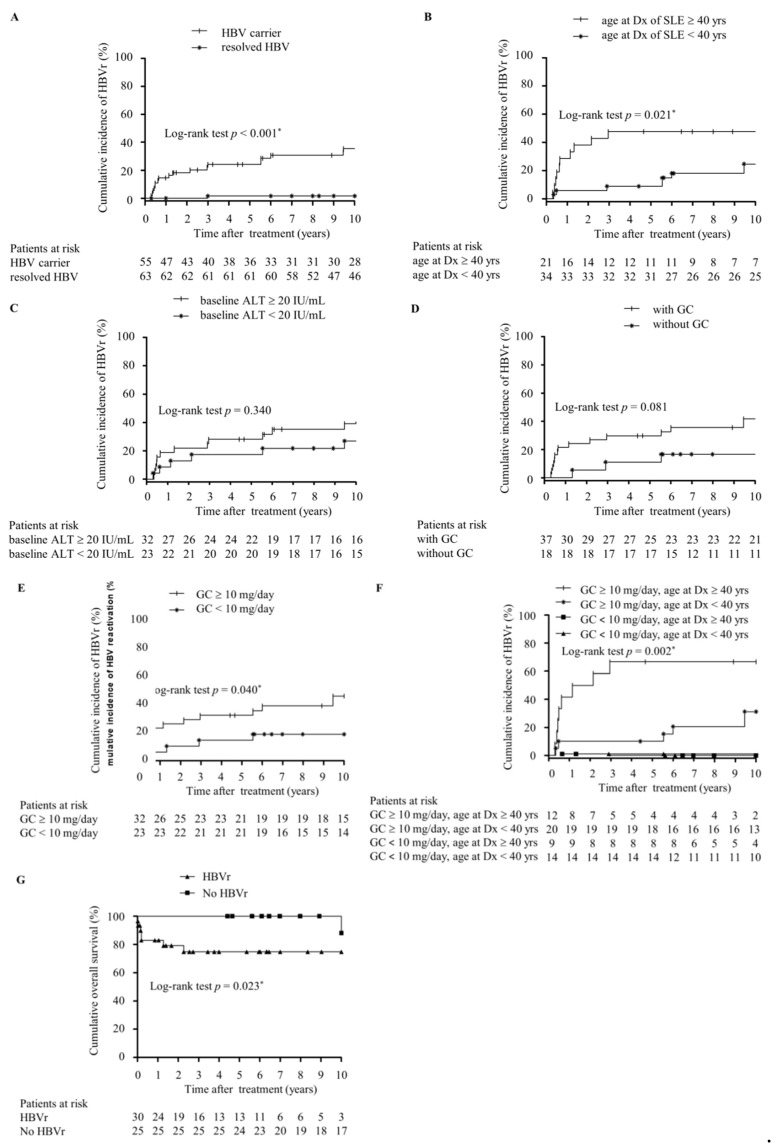
Cumulative incidence of hepatitis and HBV reactivation in SLE patients and outcome of HBsAg-positive SLE patients. (**A**) 10-year (yr) cumulative incidence of hepatitis related to HBV reactivation (HBVr) in HBsAg-positive and resolved hepatitis B SLE patients after treatment with immunosuppressive therapy. (**B**–**E**) 10-yr cumulative risk of HBVr in HBsAg-positive SLE patients stratified by the age at diagnosis (Dx) of SLE (older or younger than 40 years) (**B**), stratified by baseline serum ALT (more or less than 20 IU/mL) (**C**), stratified by with or without glucocorticoid(GC)-containing immunosuppressive therapy (**D**), stratified by with or without GC ≥ 10 mg/day prednisolone equivalents (**E**), and stratified by the age at Dx of SLE (older than 40 years with GC ≥ 10 mg/day prednisolone equivalents or not) (**F**). (**G**) 10-yr survival in 55 HBsAg-positive SLE patients with and without HBVr. The duration of follow-up was calculated from the time of treatment for SLE to the date of the last visit or death. The incidence of HBVr or mortality was evaluated by Kaplan–Meier analysis and log-rank test. *p* value < 0.05 was considered statistically significant.

After a follow-up of 1817 person years (mean 15.4 years per patient), 32 patients developed HBVr or HBsAg RS, with the incidence rate of 17.6 per 1000 person years (Figure 1). HBVr developed in 30 (54.5%) of 55 HBsAg-positive patients, with the incidence rate of 46.6 per 1000 person years. The mean time to HBVr was 8.7 years (range from 4 months to 34 years) after the start of immunosuppressants. Two (3.2%) out of 63 RHB cases experienced HBsAg RS, with the incidence rate of 1.7 per 1000 person years. The mean time to HBsAg RS was 14.7 years (8 and 21 years, respectively) after receiving immunosuppressive therapy. In RHB group, 50 were positive for anti-HBs and one (2.0%) developed HBsAg RS. Among another 13 patients negative for anti-HBs at baseline, one (7.7%) experienced HBsAg RS. SLE patients positive for HBsAg had a higher risk of HBVr compared to RHB patients (HR = 26.16, 95% CI: 6.22–110.07, *p* < 0.001, Figure 2A).

### 2.3. Clinical Features of HBVr in HBsAg-Positive SLE Patients

Among the 30 HBV carriers with reactivation, the peak HBV viral loads ranged from 3570 to >170,000,000 IU/mL, with the peak alanine aminotransferase (ALT) levels ranging from 102 to 8310 IU/mL, and the peak total bilirubin level ranged from 0.3 to 40.4 mg/dL (Table 2). Male was more common in patients with HBVr than in those without (30.0% vs. 8.0%, *p* = 0.035). There was no difference in age, baseline serum ALT, or aspartate aminotransferase (AST) levels, and immunologic profiles, including ANA titer, anti-SSA/Ro, anti-SSB/La, anti-Smith, anti-RNP, and anti-dsDNA in patients with or without reactivation. At the time of HBVr, nine (32.1%) were positive for HBeAg. Glucocorticoid with or without other immunosuppressants were more frequently prescribed in patients with HBVr than those without (25 of 30, 83.3% vs. 12 of 25, 48.0%, *p* = 0.005). In addition, more patients with HBVr received glucocorticoid ≥ 10 mg/day prednisolone equivalents than patients without HBVr (73.3% vs. 40.0%, *p* = 0.012).

In univariate analysis (Table 3), age at diagnosis of SLE older than 40 years (hazard ratio [HR] = 2.85, 95% confidence intervals [CI]: 1.26 to 6.45, *p* = 0.012), female gender (HR = 2.41, 95% CI: 1.09 to 5.31, *p* = 0.030), receiving glucocorticoid-containing immunosuppressive therapy (HR = 3.01, 95% CI: 1.15 to 7.88, *p* = 0.025), and receiving glucocorticoid ≥ 10 mg/day prednisolone equivalents (HR = 2.58, 95% CI: 1.15 to 5.82, *p* = 0.022) were associated with the risk of HBVr. In the multivariate Cox proportional hazard model, age at diagnosis of SLE older than 40 years (adjusted HR = 5.3, 95% CI: 2.12 to 13.25, *p* < 0.001, Figure 2B), baseline serum ALT levels ≥ 20 IU/mL (adjusted HR = 2.44, 95% CI: 1.07 to 5.58, *p* = 0.034, Figure 2C), and glucocorticoid-containing immunosuppressive therapy (HR = 4.78, 95% CI: 1.72 to 13.28, *p* = 0.003, Figure 2D) remained significantly associated with HBVr after adjustment of other associated factors. In addition, receiving glucocorticoid ≥ 10 mg/day prednisolone equivalents was another independent risk factor for HBVr in HBsAg-positive SLE patients (HR = 3.68, 95% CI: 1.56 to 8.65, *p* = 0.003, Figure 2E). When analyzed by age at diagnosis of SLE and dose of glucocorticoid, the risk of HBVr was highest in patients who were diagnosed with SLE at an older age (≥40 years) and received glucocorticoid ≥ 10 mg/day prednisolone equivalents (Log-rank test *p* = 0.002, Figure 2F).

### 2.4. Outcome of HBsAg-Positive SLE Patients with HBVr

HBVr-related hepatitis happened in 27 (27 out of 30, 90.0%) HBsAg-positive SLE patients with HBVr. Eleven (36.7%) patients experienced severe hepatitis (ALT >10 upper limit of normal [ULN]) during HBVr, while liver decompensation was observed in 10 (33.3%) cases. Twenty-six (86.7%) HBsAg-positive SLE patients received nucleos (t) ide analogues (NUCs) therapy at the time of HBVr, including 17 lamivudine, five entecavir, and four tenofovir treatments. Seven (23.3%) cases died of liver failure after HBVr even with prompt NUCs initiation. All patients died within three months after HBVr. Ten-year survival analysis by using the log-rank test showed that patients with HBVr had worse overall survival as compared to those without HBVr (*p* = 0.023, Figure 2G). The main difference between patients who lived or who died from HBVr in HBsAg-positive SLE patients was the peak total bilirubin levels during HBVr (*p* < 0.001) (Table S1). In the multivariate Cox proportional hazard model (Table S2), peak total bilirubin levels more than 5 mg/dL during HBVr was the only independent risk factor for mortality after adjustment for age at HBVr (adjusted OR = 110.78, 95% CI: 5.85 to 2097.68, *p* = 0.002).

### 2.5. Clinical Features of HBVr in RHB Patients

**Figure 3:**
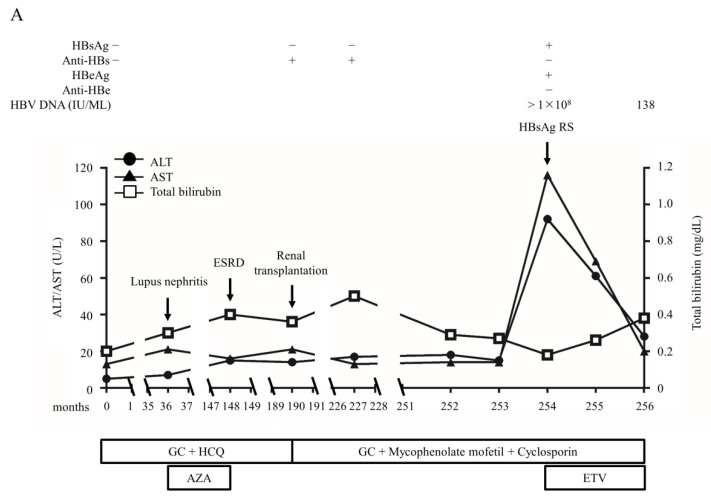

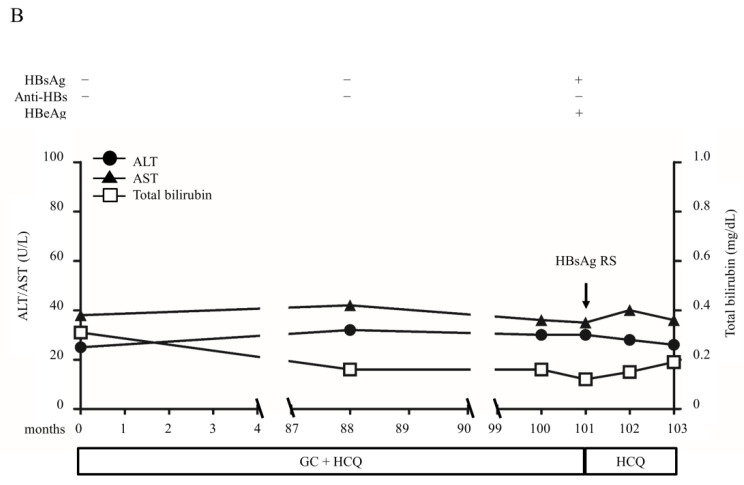
The clinical course of two resolved hepatitis B patients who developed HBsAg reverse seroconversion. (**A**) A SLE patient had positive antibodies to HBsAg (anti-HBs) titer at baseline and lupus nephritis developed three years after SLE diagnosis. She then received immunosuppressive treatment with glucocorticoids (GC), hydroxychloroquine (HCQ), and azathioprine (AZA). Unfortunately, she progressed to end-stage renal disease (ESRD) and was treated with hemodialysis 112 months later. She received a kidney transplant 42 months later and augmented immunosuppressants with GC, mycophenolate mofetil, and cyclosporin were used to prevent graft rejection. After 37 months, anti-HBs became weakly positive and HBV reactivation (HBVr) developed 27 months later with HBsAg reverse seroconversion (HBsAg RS). The peak HBV viral load was >8 log IU/mL and peak ALT level was 427 IU/mL during HBVr. The patient received emergent entecavir treatment and survived. (**B**) The patient was negative for anti-HBs before the use of immunosuppressive agents. She exposed to GC treatment for 101 months with a high accumulative GC dosage (165,768 mg prednisolone) before HBsAg RS. The peak HBV viral load was missing and peak ALT level was 142 IU/mL. She did not receive antiviral treatment because of spontaneous ALT decline and survived.

Among the 63 RHB patients, only two (3.17%) developed HBsAg RS during the follow-up. Both were female and diagnosed as SLE at the age of 20. The clinical courses of the two cases are illustrated in Figure 3. One received long-term immunosuppressive therapy for over 20 years before the HBsAg RS. She had positive anti-HBs titer at baseline (Figure 3A). Lupus nephritis developed 3 years after SLE diagnosis, and she started treatment for end stage renal disease by repeated hemodialysis 112 months later. She received a kidney transplant 42 months later and augmented immunosuppressants were used to prevent graft rejection. After 37 months, anti-HBs became weakly positive and HBVr developed 27 months later with HBsAg RS. The peak HBV viral load was > 8 log IU/mL and peak ALT level was 427 IU/mL during HBVr. The patient received emergent entecavir treatment and survived. Another patient was negative for anti-HBs before the use of immunosuppressive agents (Figure 3B). HBsAg RS developed after receiving 101 months of immunosuppressive therapy. The peak HBV viral load was missing and peak ALT level was 142 IU/mL. She did not receive NUCs treatment because of spontaneous ALT decline and survived.

## 3. Discussion

In this study, we investigated the issue of HBVr in SLE patients, which had not been previously studied well. To the best of our knowledge, the current study is the longest and largest cohort study that investigated the incidence of HBVr or HBsAg RS in SLE patients. Our findings disclosed that the incidence of HBVr was extremely high in HBsAg-positive SLE patients, and the mortality was also high after reactivation of HBV.

HBVr is a concern when hepatitis B patients are exposed to either immunosuppressive or biologic therapies for the management of rheumatologic or underlying diseases. Long-term immunosuppressive therapy is generally administrated for chronic autoimmune diseases, including SLE. Some SLE patients with multiple major organ involvement, such as kidneys, lungs, and brain, have to receive life-long immunosuppressive therapy [20,21]. Even though immunosuppressive therapy is effective at maintaining disease remission, it can impair host immune functions and increase the risk of reactivation. Among these immunosuppressive agents, we identify that glucocorticoid-containing immunosuppressive therapy is an independent risk factor for HBVr in HBsAg-positive patients. The use of moderate to high doses of glucocorticoid has been found to be associated with HBVr in SLE patients [22]. The American Gastroenterological Association (AGA) suggests that treatment with high doses of GCs (>20 mg prednisolone or equivalents) for more than four weeks should be considered as having at least moderate risk for HBVr [23]. However, the evidence regarding the risk was based on patients with chronic lung diseases; it has reported that HBVr was more frequent in patients with asthma or chronic obstructive pulmonary disease receiving medium to high (>20 mg/day) dose glucocorticoid compared with those receiving low (≤20 mg/day) dose glucocorticoid [24]. In the current study, a lower dose of glucocorticoid (≥10 mg prednisolone equivalents) was identified as an independent risk factor for HBVr in HBsAg-positive SLE patients. The immunological abnormalities in patients with SLE may be a possible explanation [25,26]. Furthermore, some immunosuppressive agents usually used in the treatment of SLE can not only induce HBVr through the suppression of the host’s immune system, but also directly cause liver damage [27,28]. Besides, we demonstrated that age older than 40 years at diagnosis of SLE significantly increased the risk of HBVr. Similarly, previous studies also demonstrated that older age is one of the key risk factors for HBVr in patients with chronic hepatitis B [29,30]. Taken together, HBsAg-positive SLE patients who were diagnosed with SLE at an older age (≥40 years) and received treatment with doses ≥ 10 mg/day of oral prednisone (or equivalent) had the highest risk of HBVr. Glucocorticoid should be avoided or the dose should be minimized in older SLE patients positive for HBsAg. Close monitoring of liver functions is necessary for HBsAg-positive SLE patients under multiple immunosuppressant treatments.

The presence of anti-HBs has been identified as a possible protective factor against HBVr [9,31]. In this study, only two RHB patients experienced HBVr and one was positive for anti-HBs before receiving immunosuppressants. It is worth noting that this patient lost anti-HBs gradually following augmented immunosuppressive therapy, which was used to prevent kidney transplant rejection. Another patient was negative for anti-HBs at baseline and exposed to glucocorticoid treatment for a long period with a high accumulative glucocorticoid dosage (165,768 mg prednisolone) before HBsAg RS. In patients under immunosuppressive therapy, an isolated anti-HBc cannot be considered a marker of HBV resolution in the absence of an anti-HBs. The titer of anti-HBs should be closely monitored in RHB SLE patients who receive immunosuppressants.

Antiviral prophylaxis by NUCs can prevent HBVr before receiving chemotherapy and immunosuppressive therapy in patients positive for HBsAg and is recommended by AASLD and EASL guidelines [32,33]. Prophylactic entecavir before rituximab-based chemotherapy has been proven to be able to prevent HBVr in lymphoma patients with RHB [9]. However, without a randomized controlled trial, it is undetermined whether antiviral prophylaxis is worth applying in HBsAg-positive SLE patients who are planning to receive immunosuppressive therapy. In addition, the cost-effectiveness of prophylactic antiviral treatment in SLE patients is a huge economic burden as these patients usually require long-term immunosuppressive therapy. As a result, there is still no reimbursement of antiviral prophylaxis for non-cancer patients in the Taiwan National Health Insurance System. This current study showed that the timing of HBVr in relation to the administration of immunosuppressive therapy was varied, indicating that serum HBV DNA and ALT should be regularly monitored after initiating immunosuppressive therapy, and antiviral prophylaxis should be administered for patients at high risk of HBVr.

Up to 23.3% of HBsAg-positive patients with HBVr expired even when NUCs were administered immediately after reactivation. There is only one previous study investigating the outcome of SLE patients with chronic hepatitis B; Thong et al. demonstrated that one out of nine (11.1%) SLE patients with HBVr died [34]. Moreover, our SLE patients with HBVr had a significantly worse prognosis than those without HBVr in overall survival. We also observed that peak total bilirubin during reactivation was associated with mortality. This finding was consistent with previous studies that reported that total bilirubin was a major predictive factor of early mortality in patients with decompensated chronic hepatitis B [35,36].

The limitations of this retrospective study are that not all SLE patients had available HBV markers, and there was no baseline HBV viral load for some patients who developed HBVr because most rheumatologists were not previously aware of the importance and the risk of HBVr. It is also the case that only 56% of oncologists are aware of antiviral prophylaxis and only 14% of them screen for HBV markers in their cancer patients [37,38]. The definition of HBV reactivation in this study was not complied with 2018 AASLD guidance [32]. This is because the patient cohorts were collected before the year 2018. Currently, the definition of HBVr was slightly different between oncologist and hepatologist. A 10-fold increase in HBV viral load is still applied by oncologists as the definition of HBVr according to the American Society of Clinical Oncology (ASCO) [39] and previous studies on rheumatic diseases [5,10,22]. Furthermore, the definition we used was even stricter than AASLD 2018; we defied HBVr as HBV DNA > 20,000 IU/mL in cases without baseline HBV viral load, while AASLD 2018 defined HBVr as HBV DNA > 10,000 IU/mL if the baseline level was not available [32]. Among the 30 patients who experienced HBVr, only one did not meet the definition of HBVr of 2018 AASLD (between 1–2 log increase in HBV DNA). In this case, the baseline HBV DNA was 116 IU/mL, with a peak HBV viral load of 4860 IU/mL, accompanied with ALT elevation to 413 IU/mL. Therefore, we classified this patient as HBVr.

## 4. Materials and Methods

### 4.1. Patients

A total of 3307 SLE patients who were diagnosed in the Taipei Veterans General Hospital and Far Eastern Memorial Hospital between January 2000 and December 2017 with a minimum three-month follow-up were retrospectively reviewed. All patients fulfilled the 1997 American College of Rheumatology classification criteria for SLE or the 2012 Systemic Lupus International Collaborating Clinics (SLICC) criteria [40,41]. The immunological profiles, clinical courses, and outcomes were recorded. Among them, 607 had available HBsAg status during diagnosis or before immunosuppressive treatment. Liver function was regularly monitored every two–three months for all cases, while HBsAg and anti-HBs status were determined bi-annually after starting immunosuppressive treatment for RHB patients. For HBsAg-positive patients, HBV DNA was measured every six months.

### 4.2. Definition

HBVr was defined as either an increase in HBV DNA > 1 Log10 IU/mL compared with baseline, or HBV DNA > 20,000 IU/mL in cases without baseline HBV viral load for HBsAg positive cases, or HBsAg RS for RHB patients [5,10,42]. Hepatitis related to HBVr was defined as at least a two-fold increase in serum ALT level as compared with the baseline level and more than three-fold higher than the ULN. The upper limit of normal for ALT is 40 U/L in this study. Other causes of liver damage, such as autoimmune hepatitis, hepatitis C virus, and hepatitis D virus co-infection had been carefully reviewed and excluded. Many drugs used for SLE have hepatotoxic potential. We had carefully reviewed the medical histories, and drug-induced liver injury (DILI) was excluded based on the clinical diagnostic scale (CDS) scoring system [43,44]. HBsAg RS in RHB patients was defined as reappearance of HBsAg in the serum. The definition of severe hepatitis was a hepatitis flare with an ALT increase to more than 10-fold ULN [45], while liver decompensation was defined as a serum total bilirubin level more than 2 mg/dL and/or prolongation of prothrombin time for more than 3 s [46,47].

### 4.3. Liver Function and Immunologic Tests

ALT, AST, and bilirubin were measured by a 24-factor automated chemical analyzer and standard reagents. The specific IgG against SSA/Ro, SSB/La, smith, and ribonucleoprotein (RNP) were quantified with an automated immunofluorescent device with a solid phase (ImmunoCAP 100, Phadia AB, Uppsala, Sweden). For the detection of anti-nuclear antibodies (ANA), an indirect immunofluorescence antibody test was used with a Fluoro-Kit™ (Diasorin, Inc., Stillwater, MI, USA). Anti-double strand DNA (dsDNA) antibody was quantified by enzyme linked immunosorben assay (ELISA) via BINDAZYME™ human kit (The Binding Site Ltd., Birmingham, UK).

### 4.4. Serological Tests of Viral Hepatitis Markers

HBsAg, Anti-HBs, HBeAg, and anti-HBc IgG were determined by radioimmunoassay (Austria-II, Ausab; Corab, Abbott Laboratories, Chicago, IL, USA) before June 2010, and quantitative chemiluminescent microparticle immunoassay (Architect i2000; Abbott Laboratories, IL, USA) since June 2010. HBV viral load was quantified by a bDNA amplification assay (Versant HBV DNA, Bayer Diagnostics, Puteaux, France) before the year 2006 and the detection limit of this assay was 2000 copies/mL. HBV DNA was determined by Roche Cobas Taqman HBV DNA assay (detection limit of 20 IU/mL, Roche Diagnostics, Basel, Switzerland).

### 4.5. Statistical Analysis

The duration of the patient’s follow-up was calculated from the time of treatment for SLE to the date of HBVr, the last visit, or death. Fisher’s exact tests were used to compare categorical variables. An independent Student’s *t*-test was used to compare numerical data following a normal distribution and the Mann–Whitney U-test was used for data violating the normal distribution. Analysis of factors for HBVr was performed using the Cox proportional hazards model. Covariates with a significance of <0.2 in the univariate logistic regression analyses were further introduced into a multivariable model with automatic backward elimination. The cumulative risk of HBVr, HBsAg RS, and overall survival were estimated using the Kaplan–Meier methods and the statistical differences were tested by log-rank tests. *p* values < 0.05 were considered statistically significant. Statistical analyses were performed using SPSS version 22.0 (IBM SPSS Statistics for Windows, IBM, Armonk, NY, USA).

## 5. Conclusions

In conclusion, HBVr was common in SLE patients who were positive for HBsAg and is associated with poor survival. Screening of hepatitis B markers, including HBsAg, anti-HBc, and anti-HBs, prior to immunosuppressive therapy is necessary for SLE patients. Our findings confirm that antiviral prophylaxis should be considered for HBsAg-positive SLE patients when starting immunosuppressive treatment, even under limited medical resources.

## Figures and Tables

**Table 1:** Demographics and hepatitis B virus status of 118 patients with SLE.

Demographics	All	HBsAg-Positive	Resolved HBV	*p*-Value
	N = 118	N = 55	N = 63	
Age at diagnosis of SLE (years)	35.2 ± 14.7	37.0 ± 15.2	33.6 ± 14.2	0.213
Female	97 (82.2)	44 (80.0)	53 (84.1)	0.559
Baseline ALT (IU/mL)	23.4 ± 12.3	25.0 ± 12.3	22.1 ± 12.2	0.200
Baseline AST (IU/mL)	24.7 ± 10.3	26.3 ± 10.5	23.4 ± 10.0	0.125
Baseline total bilirubin (mg/dL)	0.42 ± 0.19	0.45 ± 0.19	0.40 ± 0.19	0.166
HBeAg-positive	10/64 (15.6)	10/47 (21.3)	0/17 (0.0)	-
Anti-HBs positive	50 (42.4)	0 (0.0)	50 (79.4)	-
Immunologic profiles				
ANA titer > 1:80	118 (100.0)	55 (100.0)	63 (100.0)	-
Anti-SSA/Ro positive	50/94 (53.2)	17/42 (40.5)	33/52 (63.5)	0.026 *
Anti-SSB/La positive	17/89 (19.1)	5/41 (12.2)	12/48 (25.0)	0.120
Anti-Smith positive	11/78 (14.1)	2/39 (5.1)	9/39 (23.1)	0.018 *
Anti-RNP positive	23/74 (31.1)	7/36 (19.4)	16/38 (42.1)	0.028 *
Anti-dsDNA positive	105 (89.0)	47 (85.5)	58 (92.1)	0.252
Clinical manifestations				
Hematologic disorder	81 (68.6)	34 (61.8)	47 (74.6)	0.174
Kidney involvement	49 (41.5)	20 (36.4)	29 (46.0)	0.287
CNS involvement	20 (16.9)	10 (18.2)	10 (15.9)	0.739
Psychosis	4 (3.4)	2 (3.6)	2 (3.2)	0.890
Serositis	13 (11.0)	7 (12.7)	6 (14.3)	0.580
Joint involvement	86 (72.9)	37 (67.3)	49 (77.8)	0.201
Skin involvement	105 (89.0)	47 (85.5)	58 (92.1)	0.252
Follow up (years)	15.4 ± 10.3	11.7 ± 9.4	18.6 ± 10.2	<0.001 *

Abbreviations: ANA, anti-nuclear antibody; anti-HBs, antibody to hepatitis B surface antigen; CNS, central nervous system; dsDNA, double-stranded DNA; HBeAg, hepatitis B virus e antigen; HBsAg, hepatitis B surface antigen; RNP, ribonucleoprotein. Data are presented as frequency (percentage) or mean ± standard deviation. * *p* < 0.05.

**Table 2:** Characteristics of HBsAg-positive SLE patients with or without HBV reactivation.

Demographics	HBV Reactivation	No HBV Reactivation	*p*-Value
	N = 30	N = 25	
Age at diagnosis of SLE (years)	37.6 ± 16.5	36.3 ± 13.8	0.758
Female	21 (70.0)	23 (92.0)	0.035 *
Baseline ALT (IU/mL)	26.3 ± 10.8	23.4 ± 14.0	0.400
Baseline AST (IU/mL)	26.8 ± 8.9	25.7 ± 12.4	0.718
Baseline TBIL (mg/dL)	0.45 ± 0.22	0.44 ± 0.15	0.776
HBeAg positive	9/28 (32.1)	0/19 (0.0)	-
Immunosuppressive treatment			0.046 *
No treatment	3	7	
GC alone	7	3	
Immunosuppressants ^†^ alone	2	6	
GC in combination with other immunosuppressants ^†^	18	9	
Any GC containing regiments	25 (83.3)	12 (48.0)	0.005 *
Duration of GC (days)	212 (50–833)	0 (0–1522)	0.768
Cumulative GC use (gm) ^‡^	3.6 (0.5–16.4)	3.9 (0–27.5)	0.654
GC ^‡^ ≥ 10 mg/day	22 (73.3)	10 (40.0)	0.012 *
Number of immunosuppressants ^†^			0.137
1	11	11	
2	5	3	
≥3	4	1	
Peak HBV viral load during HBVr (IU/mL)	1,048,000 (41,500–13,700,000)	-	-
Peak ALT during HBVr (IU/mL)	281 (151–560)	-	-
Peak T. Bili during HBVr (mg/dL)	2.2 (0.8–4.8)	-	-
Rescue antiviral treatment	26 (86.7)	-	-

Abbreviations: ALT, alanine aminotransferase; AST, aspartate aminotransferase; GC, glucocorticoid; HBeAg, hepatitis B e antigen; HBVr, HBV reactivation; T. Bili, total bilirubin. ^†^ Immunosuppressive drugs included hydroxychloroquine, cyclophosphamide, azathioprine, mycophenolate mofetil, etc. ^‡^ Values represent prednisolone equivalents. Data are presented as frequency (percentage), mean ± standard deviation, or median (interquartile range). * *p* < 0.05.

**Table 3:** Factors associated with HBV reactivation among 55 HBsAg-positive SLE patients.

Predictors	Total (n = 55) Number (%)	HBVr (n = 30) Number (%)	Observed Period (Person-Year)	Crude HR (95% CI)	*p*-Value	Adjusted HR ^1^ (95% CI)	*p*-Value	Adjusted HR ^2^ (95% CI)	*p*-Value
Age at diagnosis of SLE									
<40 years	34 (61.82)	18 (60.00)	519.7	1.00		1.00		1.0	
≥40 years	21 (38.18)	12 (40.00)	122.2	2.85 (1.26–6.45)	0.012 *	5.30 (2.12–13.25)	<0.001 *	4.12 (1.68–10.08)	0.002 *
Gender									
Male	11 (20.00)	9 (30.00)	92.8	1.00				1.0	
Female	44 (80.00)	21 (70.00)	549.1	2.41 (1.09–5.31)	0.030 *			2.11 (0.93–4.78)	0.072
Baseline ALT									
<20 IU/mL	23 (41.82)	9 (30.00)	290.7	1.00		1.00		1.00	
≥20 IU/mL	32 (58.18)	21 (70.00)	351.2	1.80 (0.82–3.93)	0.142	2.44 (1.07–5.58)	0.034 *	2.73 (1.17–6.38)	0.020 *
Baseline AST									
<20 IU/mL	15 (27.27)	7 (23.33)	177.1	1.00					
≥20 IU/mL	40 (72.73)	23 (76.67)	464.9	1.13 (0.48–2.67)	0.775				
GC									
No	18 (32.73)	5 (16.67)	242.3	1.00		1.00			
Yes	37 (67.27)	25 (83.33)	399.6	3.01 (1.15–7.88)	0.025 *	4.78 (1.72–13.28)	0.003 *		
GC ^‡^ ≥ 10 mg/day									
No	23 (41.8)	8 (26.7)	313.7	1.00				1.00	
Yes	32 (58.2)	22 (73.3)	328.2	2.58 (1.15–5.82)	0.022 *			3.68 (1.56–8.65)	0.003 *
Drug group									
No treatment	10 (18.18)	3 (10.00)	141.2	1.00					
GC alone	10 (18.18)	7 (23.33)	79.8	3.06 (0.79–11.89)	0.107				
ISD ^†^ alone	8 (14.55)	2 (6.67)	101.1	0.93 (0.15–5.69)	0.940				
GC + ISD ^†^	27 (49.09)	18 (60.0)	319.9	2.52 (0.74–8.65)	0.141				

Abbreviations: ALT, alanine aminotransferase; AST, aspartate aminotransferase; CI, confidence interval; GC, Glucocorticoid; HbeAg, Hepatitis B e antigen; HR, hazard ratio; ISD, immunosuppressive drugs. ^†^ ISD included hydroxychloroquine, cyclophosphamide, azathioprine, mycophenolate mofetil, etc. ^‡^ Values represent prednisolone equivalents. * *p* < 0.05. ^1^ Did not include GC ≥ 10 mg/day. ^2^ Did not include GC.

## Data Availability

Not applicable.

## Supplementary Materials

The following are available online at https://www.mdpi.com/article/10.3390/ijms22179116/s1.
