# Supplementary material for: High Risk of Viral Reactivation in Hepatitis B Patients with Systemic Lupus Erythematosus
Source: Int J Mol Sci. 2021 Aug 24;22(17):9116. doi: 10.3390/ijms22179116 (PMC8430791; doi:10.3390/ijms22179116)
Supplement: Supplementary file 1 [file ijms-22-09116-s001.zip › ijms-1274943-supplementary.pdf]

Table S1. Association of variables with outcome in 30 HBsAg-positive SLE patients during HBV reactivation.

Variables	Living<br>N = 23	Liver-Related Death<br>N = 7	*p*-Value
Age at HBVr (years)	47 (25–80)	55 (25–64)	0.746
Female	16 (69.6)	5 (71.4)	1.000
Peak TBIL during reactivation (mg/dL)	1.1 (0.3–6.3)	30.4 (3.1–40.4)	<0.001 *
Peak ALT during reactivation (IU/mL)	280 (102–1015)	368 (103–8310)	0.784
Any glucocorticoid containing regiments	19 (82.6)	6 (85.7)	1.000
Anti-viral treatment, n (%)	20 (87.0)	6 (85.7)	1.000

Abbreviations: ALT, alanine aminotransferase; TBIL, total bilirubin. Data are presented as frequency (percentage), mean ± standard deviation, or median (interquartile range). * $p < 0.05$.

Table S2. Logistic-regression analysis of independent predictors of survival in 30 HBsAg-positive SLE patients during HBV reactivation.

Predictors	Total (n = 30)<br>Number (%)	Liver-Related Death (n = 7)<br>Number (%)	Crude OR<br>(95% CI)	*p*-Value	Adjusted OR † (95% CI)	*p*-Value
Age at HBVr (years)						
<55 years	21 (70.0)	3 (42.9)	1.00		1.00	
≥55 years	9 (30.0)	4 (57.1)	4.80 (0.80–28.90)	0.087	2.39 (0.12–48.00)	0.570
Gender						
Male	9 (30.0)	2 (28.6)	1.00			
Female	21 (70.0)	5 (71.4)	0.91 (0.14–5.90)	0.925		
Peak TBIL (mg/dL)						
<5 mg/dL	23 (76.7)	1 (14.3)	1.00		1.00	
≥5 mg/dL	7 (23.3)	6 (85.7)	132.0 (7.15–2435.47)	0.001 *	110.78 (5.85–2097.68)	0.002 *
Any glucocorticoid containing regiments						
No	5 (16.7)	1 (14.3)	1			
Yes	25 (83.3)	6 (85.7)	1.26 (0.12–13.59)	0.847		

Abbreviations: CI, confidence interval; OR, odds ratio; TBIL, total bilirubin. † OR and 95% CI adjusted for all other variables in the table. * $p < 0.05$.
